# Supplementary material for: Physicochemical, spectral, medicinal, and toxicological studies of Ketoprofen, Ibuprofen, and their major degradants: A quantum-chemical and in silico approach
Source: PLoS One. 2026 May 6;21(5):e0348675. doi: 10.1371/journal.pone.0348675 (PMC13148670; doi:10.1371/journal.pone.0348675)
Supplement: S1 File — S1 (a, b) Fig. Degradation pathway and IUPAC names of KTP, IBP, and their selected degradants. S1 (a, b) Table. Molecular stoichiometry of KTP, IBP, and their major degradants. S2 (a, b) Fig. HOMO-LUMO energy gaps of KTP, IBP, and their major degradants. S3 (a, b) Fig. DOS plots of KTP, IBP, and their major degradants. S2 (a, b) Table. Frontier molecular orbital (FMO) and global reactivity descriptors of KTP, IBP, and their major degradants. S4 (a, b) Fig. ESP maps of KTP, IBP, and their major degradants. S5 Fig. (a) FT-IR and (b) UV–Vis spectra (normalized) of KTP and its major degradants. S6 Fig. (a) FT-IR and (b) UV–Vis spectra (normalized) of IBP and its major degradants. S3 (a, b) Table. Selected vibrational frequencies of KTP, IBP, and their major degradants. S4 (a, b) Table. UV-Vis spectral data of KTP, IBP, and their major degradants. S5 (a, b) Table. Binding affinity and interactions of KTP, IBP, and their major degradants with the receptor protein (5F19). S7 (a, b) Fig. Nonbonding interactions and hydrogen bond surface area of IBP, and some of its degradants, with the receptor protein 5F19. S8 Fig. MD simulations over 100 ns: Radius of gyration (Rg) (a) KTP, (b) IBP; Solvent accessible surface area (SASA) (c) KTP, (d) IBP; each with KTP, IBP, and their selected degradant–5F19 complexes. S6 (a, b) Table. QSAR analysis of KTP, IBP, and their major degradants. (DOCX) [file pone.0348675.s001.docx]

Physicochemical, spectral, medicinal, and toxicological studies of Ketoprofen, Ibuprofen, and their major degradants: a quantum-chemical and in silico approach

Protyoi Chakraborty ^1, 7 ¶^ , Saithajit Mohajan ^2, 7 ¶^, Omme Samia ^3, 7^, Nusrat Jahan ^2, 7^, Nazmul Islam ^4, 7^, Mahbub Alam ^5 ¶^, Monir Uzzaman ^6, 7 ¶ *^

^1^ Department of Chemistry, School of Physical Sciences, Shahjalal University of Science and Technology, Sylhet, Bangladesh

^2^ Department of Applied Chemistry and Chemical Engineering, University of Chittagong, Chattogram, Bangladesh

^3^ Department of Pharmacy, Comilla University, Comilla, Bangladesh

^4^ Department of Chemistry, Bhawal Badre Alam Government College, Gazipur, Bangladesh

^5^ Department of Chemistry, Bangladesh Army University of Science and Technology, Khulna, Khulna, Bangladesh

^6^ Faculty of Science, Department of Chemistry, University of Chittagong, Chattogram, Bangladesh

^7^ Department of Drug Design, Computer in Chemistry and Medicine Laboratory, Dhaka, Bangladesh

^*^ Corresponding Author

Email: [monircu92@gmail.com](mailto:monircu92@gmail.com)

**Table of Contents**

| Fig S1 | Degradation pathway and IUPAC names of KTP, IBP, and their selected degradants. | S2-S3 |
| --- | --- | --- |
| Table S1 | Molecular stoichiometry of KTP, IBP, and their major degradants. | S4 |
| Fig S2 | HOMO-LUMO energy gaps of KTP, IBP, and their major degradants. | S5-S8 |
| Fig S3 | DOS plots of KTP, IBP, and their major degradants. | S8-S10 |
| Table S2 | Frontier molecular orbital (FMO) and global reactivity descriptors of KTP, IBP, and their major degradants. | S10-S11 |
| Fig S4 | Molecular electrostatic potential (MEP) maps of KTP, IBP, and their major degradants. | S11-S12 |
| Fig S5 | FT-IR and UV–Vis spectra of KTP and its major degradants. | S13 |
| Fig S6 | FT-IR and UV–Vis spectra of IBP and its major degradants. | S13 |
| Table S3 | Selected vibrational frequencies of KTP, IBP, and their major degradants. | S13-S15 |
| Table S4 | UV-vis spectral data of KTP, IBP, and their major degradants. | S15-S16 |
| Table S5 | Binding affinity and interactions of KTP, IBP, and their major degradants with the receptor protein (5F19). | S16-S20 |
| Fig S7 | Non-bonding interactions and hydrogen bond surface area of IBP, and some of its degradants with the receptor protein 5F19. | S20-S22 |
| Fig S8 | MD simulations over 100 ns: Radius of gyration (Rg) (a) KTP, (b) IBP; Solvent accessible surface area (SASA) (c) KTP, (d) IBP; each with KTP, IBP and their selected degradant–5F19 complexes. | S23 |
| Table S6 | QSAR analysis of KTP, IBP, and their major degradants. | S24-S25 |
|  | References. | S26 |

A = Casamino acid consortium-mediated KTP degradation under aerobic conditions, B = photochemical degradation, C = Photolytic degradation, D = Mineralization of KTP in aqueous medium by hydroxyl radicals

**Fig S1 (a)** Chemical structure and IUPAC name of KTP, and its selected degradants.

A = Chemical oxidation degradation, B = Hydroxy radical induced degradation, C = Benzylic oxidation, D = UV / H_2_O_2_, E = Hydrodynamic Cavitation

**Fig S1 (b)** Chemical structure and IUPAC name of IBP, and its selected degradants.

**Table S1 (a)** Molecular formula (MF), molecular weight (MW), enthalpy, free energy (Hartree), and dipole moment (Debye) of KTP, and its major degradants.

| Name | MF | MW | Enthalpy | Free Energy | Dipole Moment |
| --- | --- | --- | --- | --- | --- |
| KTP | C_16_H_14_O_3_ | 254.285 | -843.606 | -843.671 | 3.238 |
| KTP1 | C_16_H_14_O_5_ | 286.283 | -994.059 | -994.127 | 3.005 |
| KTP2 | C_16_H_14_O_4_ | 270.284 | -918.819 | -918.886 | 1.682 |
| KTP3 | C_11_H_12_O_5_ | 224.212 | -802.361 | -802.423 | 5.122 |
| KTP4 | C_14_H_10_O_3_ | 226.231 | -765.023 | -765.081 | 2.277 |
| KTP5 | C_11_H_12_O_5_ | 224.212 | -802.361 | -802.425 | 4.480 |
| KTP6 | C_13_H_10_O | 182.222 | -576.467 | -576.516 | 3.222 |
| KTP7 | C_15_H_14_O | 210.276 | -655.045 | -655.102 | 3.664 |
| KTP8 | C_15_H_12_O_2_ | 224.259 | -729.081 | -729.139 | 3.175 |
| KTP9 | C_16_H_16_O_3_ | 256.301 | -844.776 | -844.842 | 4.472 |

**Table S1 (b)** Molecular formula (MF), molecular weight (MW), enthalpy, free energy (Hartree), and dipole moment (Debye) of IBP, and its major degradants.

| Name | MF | MW | Enthalpy | Free Energy | Dipole Moment |
| --- | --- | --- | --- | --- | --- |
| IBP | C_13_H_18_O_2_ | 206.285 | -656.455 | -656.516 | 1.775 |
| IBP1 | C_12_H_16_O | 176.259 | -541.932 | -541.988 | 3.646 |
| IBP2 | C_12_H_18_O | 178.275 | -543.109 | -543.166 | 1.478 |
| IBP3 | C_13_H_16_O_3_ | 220.268 | -730.490 | -730.554 | 1.872 |
| IBP4 | C_10_H_10_O_3_ | 178.187 | -612.621 | -612.673 | 2.938 |
| IBP5 | C_13_H_18_O_3_ | 222.284 | -731.670 | -731.733 | 6.459 |
| IBP6 | C_10_H_12_O_2_ | 164.204 | -538.594 | -538.647 | 1.765 |

**Fig S2 (a)** HOMO-LUMO energy gaps of KTP, and its major degradants.

**Fig S2 (b)** HOMO-LUMO energy gaps of IBP, and its major degradants.

**Fig S3 (a)** DOS plots of KTP, and its major degradants.

**Fig S3 (b)** DOS plots of IBP, and its major degradants.

**Table S2 (a)** HOMO-LUMO, Gap, chemical hardness (η), chemical softness (*S*), potentiality (*μ*) of KTP, and its major degradants.

| Name | εHOMO | εLUMO | Gap | η | *S* | *μ* |
| --- | --- | --- | --- | --- | --- | --- |
| KTP | - 6.929 | - 2.094 | 4.835 | 2.417 | 0.206 | - 4.512 |
| KTP1 | - 6.248 | - 2.442 | 3.806 | 1.903 | 0.262 | - 4.345 |
| KTP2 | - 6.811 | - 2.484 | 4.326 | 2.163 | 0.231 | - 4.648 |
| KTP3 | - 7.244 | - 1.462 | 5.781 | 2.890 | 0.172 | - 4.353 |
| KTP4 | - 6.811 | - 2.485 | 4.326 | 2.163 | 0.231 | - 4.648 |
| KTP5 | - 7.136 | - 1.254 | 5.882 | 2.941 | 0.170 | - 4.195 |
| KTP6 | - 6.954 | - 2.079 | 4.874 | 2.437 | 0.205 | - 4.516 |
| KTP7 | - 6.832 | - 2.022 | 4.810 | 2.405 | 0.207 | - 4.427 |
| KTP8 | - 7.145 | - 2.396 | 4.749 | 2.374 | 0.210 | - 4.771 |
| KTP9 | - 6.975 | - 1.226 | 5.749 | 2.874 | 0.173 | - 4.101 |

**Table S2 (b)** HOMO-LUMO, Gap, chemical hardness (η), chemical softness (*S*), potentiality (*μ*) of IBP, and its major degradants.

| Name | εHOMO | εLUMO | Gap | η | *S* | *μ* |
| --- | --- | --- | --- | --- | --- | --- |
| IBP | - 6.646 | - 0.752 | 5.894 | 2.947 | 0.169 | - 3.699 |
| IBP1 | - 6.983 | - 1.783 | 5.200 | 2.600 | 0.192 | - 4.383 |
| IBP2 | - 6.440 | - 0.376 | 6.063 | 3.031 | 0.164 | - 3.408 |
| IBP3 | - 6.988 | - 1.960 | 5.028 | 2.514 | 0.198 | - 4.474 |
| IBP4 | - 7.336 | - 2.226 | 5.110 | 2.555 | 0.195 | - 4.781 |
| IBP5 | - 6.879 | - 0.864 | 6.014 | 3.007 | 0.166 | - 3.872 |
| IBP6 | - 6.661 | - 0.752 | 5.909 | 2.954 | 0.169 | - 3.706 |

**Fig S4 (a)** Molecular electrostatic potential (MEP) map of KTP, and its major degradants, illustrating the distribution of electron density.

**Fig S4 (b)** Molecular electrostatic potential (MEP) map of IBP, and its major degradants, illustrating the distribution of electron density.

 **
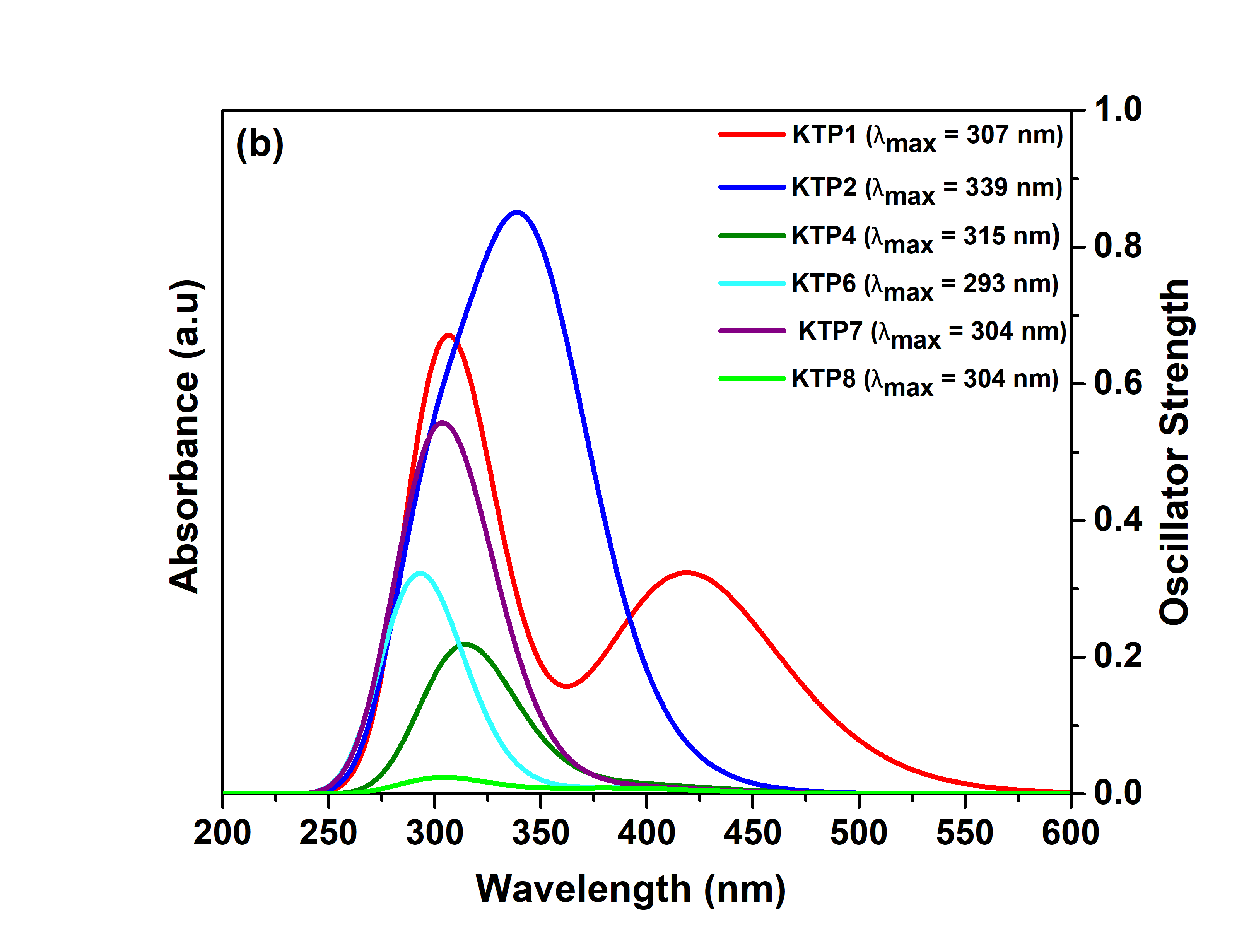
**

**Fig S5 (a)** FT-IR spectra and **(b)** UV-Vis spectra of KTP, and its major degradants.


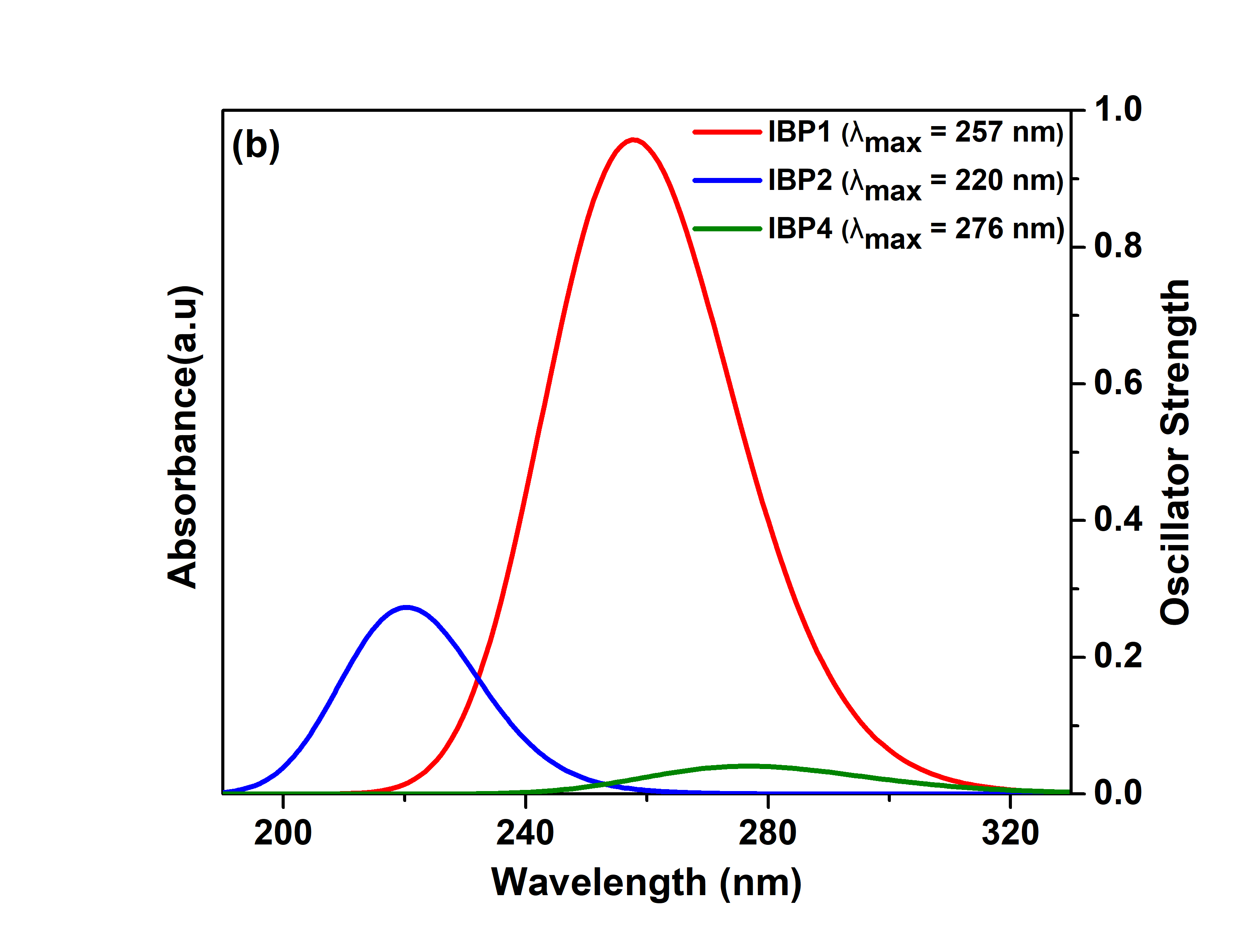


**Fig S6 (a)** FT-IR spectra **(b)** UV-Vis spectra of IBP. and its major degradants.

**Table S3 (a)** Selected vibrational frequencies of KTP, and its major degradants.

| Name | Functional group | Vibrational frequency (scaled) |
| --- | --- | --- |
| KTP | *ѵ*C-H^a^  *ѵ*C-H  *ѵ*C=C^a^  *ѵ*O-H  *ѵ*O=C | 3091 |
|  |  | 2946 |
|  |  | 1588 |
|  |  | 3614 |
|  |  | 1748 |
| KTP1 | *ѵ*C-H^a^  *ѵ*C-H  *ѵ*C=C^a^  *ѵ*O-H  *ѵ*O=C | 3086 |
|  |  | 2945 |
|  |  | 1582 |
|  |  | 3121 |
|  |  | 1611 |
| KTP2 | *ѵ*C-H^a^  *ѵ*C-H  *ѵ*C=C^a^  *ѵ*O-H  *ѵ*O=C | 3093 |
|  |  | 2951 |
|  |  | 1593 |
|  |  | 3591 |
|  |  | 1740 |
| KTP3 | *ѵ*C-H^a^  *ѵ*C-H  *ѵ*C=C^a^  *ѵ*O-H  *ѵ*O=C | 3093 |
|  |  | 2951 |
|  |  | 1593 |
|  |  | 3591 |
|  |  | 1740 |
| KTP4 | *ѵ*C-H^a^  *ѵ*C=C^a^  *ѵ*O-H  *ѵ*O=C | 3104 |
|  |  | 1588 |
|  |  | 3672 |
|  |  | 1660 |
| KTP5 | *ѵ*C-H^a^  *ѵ*C-H  *ѵ*C=C^a^  *ѵ*O-H  *ѵ*O=C | 3091 |
|  |  | 2948 |
|  |  | 1577 |
|  |  | 3617 |
|  |  | 1749 |
| KTP6 | *ѵ*C-H^a^  *ѵ*C=C^a^  *ѵ*O=C | 3089 |
|  |  | 1589 |
|  |  | 1658 |
| KTP7 | *ѵ*C-H^a^  *ѵ*C-H  *ѵ*C=C^a^  *ѵ*O=C | 3089 |
|  |  | 2929 |
|  |  | 1570 |
|  |  | 1656 |
| KTP8 | *ѵ*C-H^a^  *ѵ*C-H  *ѵ*C=C^a^  *ѵ*O=C | 3089 |
|  |  | 2939 |
|  |  | 1585 |
|  |  | 1659 |
| KTP9 | *ѵ*C-H^a^  *ѵ*C-H | 3092 |
|  |  | 2952 |
|  | *ѵ*C=C^a^  *ѵ*O-H  *ѵ*O=C | 1594 |
|  |  | 3622 |
|  |  | 1777 |

**Table S3 (b)** Selected vibrational frequencies of IBP, and its major degradants.

| Name | Functional group | Vibrational frequency (scaled) |
| --- | --- | --- |
| IBP | *ѵ*C-H^a^  *ѵ*C-H  *ѵ*C=C^a^  *ѵ*O-H  *ѵ*O=C | 3076 |
|  |  | 2929 |
|  |  | 1560 |
|  |  | 3617 |
|  |  | 1744 |
| IBP1 | *ѵ*C-H^a^  *ѵ*C-H  *ѵ*C=C^a^  *ѵ*O=C | 3064 |
|  |  | 2925 |
|  |  | 1595 |
|  |  | 1686 |
| IBP2 | *ѵ*C-H^a^  *ѵ*C-H  *ѵ*C=C^a^  *ѵ*O-H | 3070  2923  1563  3691 |
| IBP3 | *ѵ*C-H^a^  *ѵ*C-H  *ѵ*C=C^a^  *ѵ*O-H  *ѵ*O=C | 3070  2947  1593  3616  1682 |
| IBP4 | *ѵ*C-H^a^  *ѵ*C-H  *ѵ*C=C^a^  *ѵ*O-H  *ѵ*O=C | 3074 |
|  |  | 2947 |
|  |  | 1594 |
|  |  | 3615 |
|  |  | 1710 |
| IBP5 | *ѵ*C-H^a^  *ѵ*C-H  *ѵ*C=C^a^  *ѵ*O-H  *ѵ*O=C | 3059 |
|  |  | 2930 |
|  |  | 1601 |
|  |  | 3614 |
|  |  | 1773 |
| IBP6 | *ѵ*C-H^a^  *ѵ*C-H  *ѵ*C=C^a^  *ѵ*O-H  *ѵ*O=C | 3074 |
|  |  | 2926 |
|  |  | 1603 |
|  |  | 3618 |
|  |  | 1744 |

Here, a = aromatic

**Table S4 (a)** Electronic absorption of IBP, and its major degradants. Calculated at TD-DFT/6-311 g+ (d, p) level of theory.

| Name | Excited state | Wavelength  (nm) | Excitation Energy  (eV) | Configuration Composition | Oscillator Strength |
| --- | --- | --- | --- | --- | --- |
| IBP | S_0_→S_1_ | 292.30 | 4.241 | (-0.378) H-2→L, (0.579) H→L | 0.061 |
| IBP1 | S_0_→S_1_ | 351.87 | 3.523 | (0.695) H→L | 0.000 |
| IBP2 | S_0_→S_1_ | 244.27 | 5.076 | (-0.312) H-1→L, (0.294) H-1→L+1, (0.383) H→L, (0.40) H→L+1 | 0.001 |
| IBP3 | S_0_→S_1_ | 367.95 | 3.370 | (0.657) H→L, (-0.233) H→L+1 | 0.000 |
| IBP4 | S_0_→S_1_ | 359.42 | 3.449 | (0.692) H→L, (-0.108) H→L+6 | 0.000 |
| IBP5 | S_0_→S_1_ | 290.81 | 4.263 | (-0.343) H-3→L, (-0.173) H-1→L, (0.579) H→L | 0.004 |
| IBP6 | S_0_→S_1_ | 291.80 | 4.249 | (-0.381) H-2→L, (0.578) H→L | 0.055 |

**Table S4 (b)** Electronic absorption of KTP, and its major degradants. Calculated at TD-DFT/6-311 g+ (d, p) level of theory.

| Name | Excited state | Wavelength  (nm) | Excitation Energy  (eV) | Configuration Composition | Oscillator Strength |
| --- | --- | --- | --- | --- | --- |
| KTP | S_0_→S_1_ | 391.62 | 3.166 | (0.162) H-4→L, (0.216) H-3→L,  (-0.156) H-1→L, (0.618) H→L | 0.000 |
| KTP1 | S_0_→S_1_ | 419.91 | 2.952 | (0.703) H→L | 0.019 |
| KTP2 | S_0_→S_1_ | 396.10 | 3.130 | (-0.185) H-4→L, (0.241) H-2→L,  (0.523) H-1→L, (0.343) H→L | 0.001 |
| KTP3 | S_0_→S_1_ | 312.18 | 3.971 | (-0.172) H-4→L, (-0.304) H-3→L, (0.192) H-2→L, (-0.131) H-1→L, (0.560) H→L | 0.002 |
| KTP4 | S_0_→S_1_ | 395.96 | 3.131 | (0.105) H-5→L, (-0.293) H-2→L,  (0.100) H-2→L+1, (0.163) H-1→L, (0.572) H→L, (-0.153) H→L+1 | 0.000 |
| KTP5 | S_0_→S_1_ | 293.65 | 4.222 | 0.426) H-3→L, (-0.116) H-3→L+1, (0.264) H-2→L, (0.158) H-1→L, (0.395) H→L, (-0.104) H→L+1 | 0.026 |
| KTP6 | S_0_→S_1_ | 391.97 | 3.163 | (0.229) H-4→L, (-0.197) H-1→L,  (0.632) H→L | 0.000 |
| KTP7 | S_0_→S_1_ | 392.87 | 3.156 | (0.186) H-4→L, (0.171) H-3→L,  (0.301) H-1→L, (0.579) H→L | 0.000 |
| KTP8 | S_0_→S_1_ | 395.46 | 3.135 | (0.240) H-4→L, (-0.125) H-2→L,  (0.608) H→L, (-0.136) H→L+1 | 0.000 |
| KTP9 | S_0_→S_1_ | 292.81 | 4.234 | (-0.433) H-4→L, (0.192) H-2→L,  (0.457) H-1→L, (0.121) H→L | 0.025 |

**Table S5 (a)** Binding affinity and interactions of KTP, and its major degradants with the receptor protein (5F19) after rigid docking.

| Name | Residues in contact | Interaction types | Distance (Å) |
| --- | --- | --- | --- |
| KTP | SER353  TYR355  VAL349  LEU352  VAL523  ALA527  ALA527  LEU531 | C  PDH  PA  PA  PA  PA  PA  PA | 2.74590  3.33506  5.05503  4.67692  4.71481  4.94632  4.62109  5.47296 |
| KTP1 | ASN43  ARG44  ARG44  TYR130  LYS137  LYS137  CYS41  GLY45  ARG44  VAL46  PRO153  LEU152  ARG469 | CH  CH  CH  CH  CH  CH  CH  CH  C  PA  PA  PA  PA | 2.78587  2.58046  1.82818  2.08698  2.808  2.952  1.961  2.907  2.797  5.140  4.394  4.971  5.368 |
| KTP2 | ALA527  GLY533  MET522  GLY526  GLY533  PHE529  PHE381  LEU384  TYR385  TRP387  VAL349  ALA527  LEU534 | CH  CH  CH  C  C  P  PPTS  A  PA  PA  PA  PA  PA | 2.809  2.262  2.425  2.663  2.828  4.190  5.086  5.071  4.765  5.092  4.509  5.251  4.542 |
| KTP3 | LEU531  PHE529  TYR385  VAL523  GLY526  PHE529  GLY526: ALA527  LEU384  TYR385  TRP387  VAL349  ALA527 | CH  CH  CH  CH  C  P  APS  A  PA  PA  PA  PA | 2.465  2.556  2.278  2.950  2.583  4.797  4.351  5.167  4.463  5.148  4.508  4.820 |
| KTP4 | GLY526: ALA527  VAL349  LEU352 | APS  PA  PA | 4.969  5.238  4.692 |
| KTP5 | CYS41  ASN43  CYS47  GLU465  CYS41  GLY45  HIS39  ARG469  GLU465  PRO153  HIS39  LEU152 | CH  CH  CH  CH  CH  CH  C  C  C  A  PA  PA | 2.467  2.395  2.139  1.962  2.001  1.910  2.941  3.072  2.609  4.791  4.492  4.895 |
| KTP6 | LEU531  PHE381  LEU534 | CH  PPTS  PA | 2.148  5.106  4.518 |
| KTP7 | TYR130  TYR130  LEU152  VAL46  PRO153 | PDH  PPTS  PA  PA  PA | 2.895  5.050  4.725  5.416  5.427 |
| KTP8 | HIS39  GLN461  PRO156  CYS47  CYS36  PRO156  CYS41  CYS47  PRO153 | CH  CH  C  PDH  PA  PA  PA  PA  PA | 2.495  2.876  2.822  3.319  4.499  4.725  5.100  4.344  5.234 |
| KTP9 | TYR385  GLY526  PHE529  PHE529  PHE381  VAL349  LEU352  VAL349  ALA527  LEU531  LEU534 | CH  C  P  P  PPTS  A  A  PA  PA  PA  PA | 2.115  2.670  4.928  4.168  5.156  4.653  4.137  4.091  5.278  5.401  4.398 |

**Table S5 (b)** Binding affinity and interactions of IBP, and its major degradants with the receptor protein (5F19) after rigid docking.

| Name | Residues in contact | Interaction types | Distance (Å) |
| --- | --- | --- | --- |
| IBP | CYS41  HIS39  PRO40  PRO153  PRO156  CYS47  PRO153 | CH  C  C  A  A  PA  PA | 2.277  2.783  2.810  4.805  4.525  4.819  4.145 |
| IBP1 | LEU531  LEU531  GLY533  LEU534  GLY533  PHE381  VAL349  LEU352  LEU534 | CH  CH  CH  CH  C  PPS  A  A  PA | 2.804  2.754  2.765  2.647  2.937  5.869  4.662  5.110  5.108 |
| IBP2 | ASN382  ASN382  HIS207  LEU390  HIS207  PHE210  HIS386 | CH  CH  PPTS  A PA  PA  PA | 2.364  2.157  4.622  4.954  4.173  5.039  4.645 |
| IBP3 | LEU531  VAL523  TYR385  LEU352  LEU534  PHE205  PHE205  PHE209  PHE381  TYR385  PHE518  VAL349 | CH  C  PPTS  A  A  PA  PA  PA  PA  PA  PA  PA | 2.074  2.411  5.247  3.969  4.040  5.186  4.721  5.435  5.205  4.689  4.956  5.442 |
| IBP4 | ALA527  PHE529  ALA527  PHE381  VAL344  VAL349  TYR348  LEU534 | CH  CH  C  PPTS  A  A  PA  PA | 2.867  2.003  2.446  5.162  5.469  4.760  5.007  4.390 |
| IBP5 | MET522  VAL523  ALA527  VAL349  VAL523  LEU534  PHE205  PHE381  PHE381  TYR385  VAL349  ALA527 | CH  CH  A  A  A  A  PA  PA  PA  PA  PA  PA | 2.828  2.868  3.756  4.967  4.722  4.131  5.331  5.388  5.087  4.918  3.831  4.950 |
| IBP6 | LEU53  LEU531  GLY526  PHE529  PHE209  PHE381  VAL344  VAL349  TYR348  LEU534 | CH  CH  CH  P  PS  PPTS  A  A PA  PA | 2.779  2.571  2.077  4.113  2.730  5.098  5.404  4.797  5.030  4.450 |

C = Carbon hydrogen bond, A = Alkyl, PA = Pi-Alkyl, P = Pi-anion, X = Halogen bond, PS = Pi-sigma, PPS = Pi-Pi stacked, PPTS = Pi-Pi T-shaped, PDH=Pi-Donor Hydrogen Bond, CH=Conventional hydrogen bond, APS= Amide-Pi Stacked.

**Fig S7 (a)** Non-bonding interactions and hydrogen bond surface area of some of the KTP degradants with the receptor protein 5F19.

**Fig S7 (b)** Non-bonding interactions and hydrogen bond surface area of IBP, and some of its degradants with the receptor protein 5F19.

**Fig S8** MD simulations over 100 ns: Radius of gyration (Rg) (a) KTP, (b) IBP; Solvent accessible surface area (SASA) (c) KTP, (d) IBP; each with KTP, IBP and their selected degradant–5F19 complexes.

**Table S6 (a)** Quantitative structure-activity relationship study of KTP, and its degradants.

| Name | Chiv5 | bcutm1 | MRVSA9 | MRVSA6 | PEOEVSA5 | GATSv4 | J | Diametert | pIC_50_ |
| --- | --- | --- | --- | --- | --- | --- | --- | --- | --- |
| KTP | 1.206 | 3.915 | 11.753 | 71.288 | 48.531 | 1.188 | 1.885 | 10.0 | 4.529 |
| KTP1 | 1.259 | 3.933 | 11.753 | 59.155 | 24.265 | 1.086 | 1.965 | 10.0 | 4.470 |
| KTP2 | 1.233 | 3.922 | 11.753 | 65.221 | 30.332 | 1.142 | 1.904 | 10.0 | 4.451 |
| KTP3 | 0.803 | 3.873 | 11.939 | 35.392 | 24.265 | 1.148 | 2.529 | 8.0 | 4.321 |
| KTP4 | 1.006 | 3.909 | 11.753 | 71.288 | 42.465 | 1.295 | 1.868 | 9.0 | 4.348 |
| KTP5 | 0.803 | 3.873 | 11.939 | 35.392 | 24.265 | 1.148 | 2.529 | 8.0 | 4.321 |
| KTP6 | 0.863 | 3.884 | 5.783 | 71.791 | 60.664 | 2.000 | 1.818 | 8.0 | 4.342 |
| KTP7 | 1.110 | 3.903 | 5.783 | 71.288 | 55.455 | 1.778 | 1.845 | 9.0 | 4.496 |
| KTP8 | 1.076 | 3.910 | 11.566 | 71.288 | 48.531 | 1.360 | 1.868 | 9.0 | 4.394 |
| KTP9 | 1.303 | 3.908 | 5.969 | 71.288 | 54.597 | 1.188 | 1.885 | 10.0 | 4.705 |

**Table S6 (b)** Quantitative structure-activity relationship study of IBP, and its degradants.

| Name | Chiv5 | bcutm1 | MRVSA9 | MRVSA6 | PEOEVSA5 | GATSv4 | J | Diametert | pIC_50_ |
| --- | --- | --- | --- | --- | --- | --- | --- | --- | --- |
| IBP | 0.925 | 3.861 | 5.969 | 35.392 | 38.113 | 1.010 | 2.291 | 9.0 | 4.604 |
| IBP1 | 0.799 | 3.853 | 5.783 | 35.392 | 38.113 | 1.083 | 2.224 | 8.0 | 4.471 |
| IBP2 | 0.838 | 3.849 | 0.000 | 35.392 | 38.113 | 1.083 | 2.224 | 8.0 | 4.603 |
| IBP3 | 0.820 | 3.876 | 11.753 | 35.392 | 38.113 | 1.026 | 2.392 | 9.0 | 4.499 |
| IBP4 | 0.599 | 3.855 | 12.255 | 35.392 | 24.265 | 1.300 | 2.317 | 8.0 | 4.190 |
| IBP5 | 0.932 | 3.866 | 5.969 | 35.392 | 24.265 | 1.026 | 2.340 | 9.0 | 4.528 |
| IBP6 | 0.641 | 3.845 | 5.969 | 35.392 | 29.829 | 1.320 | 2.336 | 7.0 | 4.280 |

**References:**

1. Song W, Lu H, Li Q, Wang X, Fu Z, Zhou J. Aerobic degradation of ketoprofen by marine consortia: Fenton-like reaction and degradation pathway. Science of The Total Environment. 2023;892: 164520. doi:10.1016/j.scitotenv.2023.164520

2. Aguilar J, Moctezuma E, Rodríguez-Varela M, Martínez-Richa A, Vega-Rodríguez S, Leyva E. Photocatalytic degradation of non-steroidal anti-inflammatory drugs with ketoprofen as model compound. Intermediates and total reaction mechanism. J Photochem Photobiol A Chem. 2025;458: 115974. doi:10.1016/j.jphotochem.2024.115974

3. Salgado R, Pereira VJ, Carvalho G, Soeiro R, Gaffney V, Almeida C, et al. Photodegradation kinetics and transformation products of ketoprofen, diclofenac and atenolol in pure water and treated wastewater. J Hazard Mater. 2013;244–245: 516–527. doi:10.1016/j.jhazmat.2012.10.039

4. Feng L, Oturan N, van Hullebusch ED, Esposito G, Oturan MA. Degradation of anti-inflammatory drug ketoprofen by electro-oxidation: comparison of electro-Fenton and anodic oxidation processes. Environmental Science and Pollution Research. 2014;21: 8406–8416. doi:10.1007/s11356-014-2774-2

5. Sabri N, Hanna K, Yargeau V. Chemical oxidation of ibuprofen in the presence of iron species at near neutral pH. Science of The Total Environment. 2012;427–428: 382–389. doi:10.1016/j.scitotenv.2012.04.034

6. Illés E, Takács E, Dombi A, Gajda-Schrantz K, Rácz G, Gonter K, et al. Hydroxyl radical induced degradation of ibuprofen. Science of The Total Environment. 2013;447: 286–292. doi:10.1016/j.scitotenv.2013.01.007

7. Ding Z, Zhang J, Fang T, Zhou G, Tang X, Wang Y, et al. New insights into the degradation mechanism of ibuprofen in the UV/H _2_ O _2_ process: role of natural dissolved matter in hydrogen transfer reactions. Physical Chemistry Chemical Physics. 2023;25: 30687–30696. doi:10.1039/D3CP03305H

8. Musmarra D, Prisciandaro M, Capocelli M, Karatza D, Iovino P, Canzano S, et al. Degradation of ibuprofen by hydrodynamic cavitation: Reaction pathways and effect of operational parameters. Ultrason Sonochem. 2016;29: 76–83. doi:10.1016/j.ultsonch.2015.09.002
