# Supplementary material for: Physicochemical, spectral, medicinal, and toxicological studies of Ketoprofen, Ibuprofen, and their major degradants: A quantum-chemical and in silico approach
Source: PLoS One. 2026 May 6;21(5):e0348675. doi: 10.1371/journal.pone.0348675 (PMC13148670; doi:10.1371/journal.pone.0348675)
Supplement: Attachment — Submitted filename: Response to reviewers.docx [file pone.0348675.s003.docx]

**Response to reviewers**

We are thankful to the honorable reviewers for their valuable comments. We went through the comments, corrected them accordingly, and highlighted them in yellow.

**Additional Editor:**

1. Introduction needs revision focusing on the degradation product biological response

**Response**: Thanks to the reviewers for this suggestion. Corrected accordingly in the Introduction section, section 1.

2. In Abstract details regarding binding affinity and HOMO/LUMO values are missing

**Response**: Corrected accordingly in the Abstract section.

3. The article lacks the method of degradation to conduct the FTIR

**Response**: We clarified this point in the methodology section. Specifically, FT-IR spectra were generated computationally via DFT vibrational frequency calculations, and therefore, no laboratory-based degradation experiment or sample preparation was required.

4. Mention x, y z coordinates

**Response**: We have mentioned the coordinates accordingly under section 2.3.

5. Justify the selection of PDB Id?

**Response**: PDB ID: 5F19 was selected because it provides a high-resolution (2.04 Å) crystal structure of aspirin-acetylated human COX-2, which is directly involved in the inflammatory cyclooxygenase pathway. The presence of a co-crystallized aspirin molecule clearly defines the active binding site, improving docking accuracy. Additionally, chain A was used for docking, and no missing residues were present in the binding pocket region, confirming its suitability for the study. This has been included in the manuscript under section 2.3.

6. Improve the visualization of the docking poses.

**Response**: Improved accordingly in Fig 7.

7. Conclusion needs improvement

**Response**: We revised the Conclusion (Section 4) to present a clear summary of the results and the future implications of this study.

**Reviewer #1:**

1. What is the source of degradants? The source and origin of the degradants have not been clearly described.

**Response**: We have clarified the origin of the degradants in the Introduction (Section 1). The degradants were selected based on previously published experimental degradation studies (e.g., photocatalysis, UV/H_2_O_2_, electro-Fenton/anodic oxidation, cavitation, and microbial/aerobic degradation). The chemical identities and structures were taken from these literature-reported pathways and then used for the computational analyses in this manuscript.

2. Introduction requires a stronger scientific rationale with clearly stated objectives and and a brief outline of the findings of the study.

**Response**: We revised the Introduction. We added the study rationale, objectives, and summary of findings.

3. The FTIR, UV, and IC50 information was provided in the result section however, their experimental procedures were not given in the methodology section.

**Response**: We have revised and clarified the **Materials and Methods** section. FT-IR spectra are now clearly described as obtained from **DFT vibrational frequency calculations**, and UV–Vis spectra from **TD-DFT calculations at the same level of theory** (Section 2.2). The p**IC_50_ values** are explained as computational predictions derived from a **QSAR-based MLR model**, with descriptors and the regression equation provided (Sections 2.6 and 3.10). No experimental IC₅₀ measurements were performed in this study.

4. The conclusion section was not clear and it lacks to establish the study's implication for future use or research.

**Response**: We have revised the Conclusion. We clarified the findings and added their scientific implications and future relevance.

5. Is the manuscript presented in an intelligible fashion and written in standard English?

**Response**: We have revised the manuscript to improve clarity and grammar. The language has been corrected throughout. These language edits were not individually highlighted to maintain readability of the tracked changes file.
