# Supplementary material for: Physicochemical, spectral, medicinal, and toxicological studies of Ketoprofen, Ibuprofen, and their major degradants: A quantum-chemical and in silico approach
Source: PLoS One. 2026 May 6;21(5):e0348675. doi: 10.1371/journal.pone.0348675 (PMC13148670; doi:10.1371/journal.pone.0348675)
Supplement: Attachment — Submitted filename: Response to reviewers r2.docx [file pone.0348675.s004.docx]

**Response to reviewers**

We are thankful to the honorable reviewers for their valuable comments. We went through the comments, corrected accordingly, and highlighted in yellow.

•Abstract is too lengthy; it should be revise to make it simple and summarize up to 300 words highlighting main outcomes of the study only!

**Response**: Corrected accordingly in the Abstract section.

•Very old references of year 2010 and earlier (even earlier of 1990s) are there; they should be replaced with recent references.

**Response**: We have included recent and relevant references as suggested.

•It is better to mention the names of specified NSAIDs in conclusion as mentioned in title of manuscript! Further, what you have mentioned as "conclusion" may be shifted to result and discussion part while rewrite conclusion in simple 4-5 lines with final outcome of this study and future recommendations only!

**Response**: Corrected accordingly in the Conclusion section.

•A boil egg diagram will better explain kinetic details of compounds specially for BBB and GI Absorption profiling!

**Response**: Thanks to the reviewer for this suggestion. We have included boil egg diagrams in Figs 8 (a) and (b). We have also explained them under the section 3.8.

•In figures 8, proper labelling of figures 8c-8f is missing! while figure 7 and figure 8 must needs improved resolutions.

**Response**: Corrected accordingly. However, figure 8 has been replaced and **the current figure number for fig 8 is fig 9**.
